# Risk and resilience: a mixed methods investigation of Aboriginal Australian women’s perinatal mental health screening assessments

**DOI:** 10.1007/s00127-020-01986-7

**Published:** 2020-11-23

**Authors:** Emma Carlin, Kimberley H. Seear, Katherine Ferrari, Erica Spry, David Atkinson, Julia V. Marley

**Affiliations:** 1 The Rural Clinical School of Western Australia, The University of Western Australia, Broome, WA Australia; 2 Kimberley Aboriginal Medical Services, Broome, WA Australia

**Keywords:** Aboriginal, Indigenous, Perinatal, Mental health, Protective factors, Resiliency

## Abstract

**Purpose:**

To describe the psychosocial protective and risk factors for perinatal mental health identified in a sample of Aboriginal women’s Kimberley Mum’s Mood Scale (KMMS) assessments and explore the role of these factors in their screening assessment and diagnostic outcome.

**Methods:**

We used a mixed methods approach to retrospectively analyse a cross-sectional study dataset of 91 completed KMMS assessments. This included: categorising the clinical notes from the KMMS psychosocial yarn into ‘risk’ and ‘protective’ factors and describing these categories, describing the number and type of risk and protective factors associated with different KMMS risk assessment categories (no, low, medium, high), and exploring relationships between these risk and protective factors and diagnosis of perinatal depression and/or anxiety.

**Results:**

Protective factors were recorded for the vast majority of the women; the most prominent was positive family relationships. When protective and risk factors were stratified by KMMS risk category, women in the higher risk group less commonly had specific protective factors (11–33% high vs 61–100% no risk) and more commonly had risk factors (22–67% high vs 6–28% no risk) than women with lower KMMS assessed risk. The average number of protective factors decreased with increasing KMMS risk category (4.9 ± 1.1 to 1.6 ± 1.3), with the inverse pattern for risk factors (1.1 ± 1.1 to 3.8 ± 1.0). Having protective factors also appeared to reduce the risk of developing clinical depression or anxiety.

**Conclusion:**

Assessing protective factors in mental health screening for perinatal Aboriginal women increases the effectiveness of screening and provides a foundation for the delivery of local structured psychosocial care.

## Background

Perinatal mental health disorders are a major contributor to the burden of disease and disability worldwide [1–3], adversely impacting maternal quality of life and levels of productivity [4, 5]. Perinatal mental ill-health may also negatively impact birth outcomes (including preterm births and lower birth weight); maternal-infant bonding and attachment; and contribute to ongoing emotional and cognitive difficulties for the child [6]. Studies suggest 10–65% of perinatal women are affected by perinatal mental health disorders [1]. The large variation in prevalence can, in part, be understood by the non-standardised definitions of perinatal mental health disorders and associated diagnostic criteria [1, 3].

Variance in prevalence is also underpinned by the uneven distribution of perinatal mental ill-health across different population groups [1, 3, 7]. Within Australia, Aboriginal women have significantly higher rates of anxiety or depression in the perinatal period [8–10] than the non-Aboriginal perinatal population [11]. A large Australian study found 19% of Aboriginal and Torres Strait Islander women had antenatal depression versus approximately 9% among non-Indigenous women [12]. Perinatal depression and anxiety was identified in 25% of a sample of 91 perinatal Aboriginal Australian women from the Kimberley region [13].

It is unlikely to be culture or ethnicity per se that leads to elevated rates of perinatal depression, but rather that certain cultural groups experience higher exposure to multiple risk factors which are correlated with perinatal mental ill health [14]. For Indigenous women such as Aboriginal Australian women colonisation, marginalisation, dispossession and racism create environments in which clusters of risk factors form [15–18]*.* International Indigenous experiences of perinatal mental ill health emphasise cultural disconnectedness, sociodemographic vulnerability, family and/or intimate partner violence, intergenerational trauma, limited social supports, substance abuse and limited expressions of self-efficacy [14, 19]. Despite high levels of risk factors [20], most Indigenous women also have protective factors which may reduce their risk of developing perinatal mental health disorders. There is a paucity of knowledge, however, about the role these protective factors play in Indigenous women’s overall perinatal mental health [14, 19].

Understanding what supports and enables good Indigenous perinatal mental health offers unique opportunities for individuals, communities and services to mobilise and enhance perinatal wellbeing. Studies identifying the types and impact of protective factors for Indigenous peoples’ mental health, [21–28] including Indigenous perinatal mental health [29, 30], are framed by concepts of resiliency. Resiliency is defined as the ability of people to cope in the event of significant adversity or risk [31–33] and is understood as a complex interplay between an individual’s biology, psychology and social environment. Within this interplay, expressions of hope and self-efficacy, alongside perceptions of coping, control and competence, are better predictors of individual resilience than variables such as education, income or employment [31–34]. Indigenous-centred resiliency literature from Australia, New Zealand, Canada and the United States promotes the concept of a person’s health and wellbeing as holistic and optimised when connection to land, culture and family is strong and present [20, 24, 35].

In Australia, perinatal mental health screening is a component of clinical care, screening commonly occurs using the Edinburgh Postnatal Depression Scale (EPDS) [11]. The EPDS [36] is a traditional screening tool for depression and anxiety; it comprises of a series of discrete questions with prescribed responses of which women choose one. The EPDS does not assess for, or enquire about, a woman’s protective factors. There have been longstanding concerns with the suitability of the EPDS for use with Aboriginal Australian women [18] and in the Kimberley region this led to the development of the Kimberley Mum’s Mood Scale (KMMS). The KMMS is a two part perinatal depression and anxiety risk assessment tool. Part 1 of the KMMS adapts the EPDS using language and graphics determined via a community co-design process [37]. Part 2 involves the narrative approach of ‘yarning’ [38–41] as a method for health professionals to explore selected psychosocial risk and protective factors with women [37]. Women’s responses to Part 1 and Part 2 are interpreted by the health professional to determine the woman’s overall risk of depression and/or anxiety and determine next steps in her clinical/psychosocial care.

Results from the KMMS validation study demonstrated that the KMMS is capable of identifying women with moderate or high risk of depression and/or anxiety when assessed against a blinded standardised diagnostic interview conducted by a general practitioner (GP) trained and experienced in mental health assessment (sensitivity 83%; specificity, 87%; positive predictive value, 68%) [13]. This study also demonstrated that the KMMS was acceptable to women and their health professionals [13]. The KMMS validation study and subsequent consultations with other groups of Aboriginal women have identified that the inclusion of protective factors is central to Aboriginal women identifying the KMMS as acceptable and culturally safe [42, 43].

The aim of this paper is to describe the psychosocial protective and risk factors identified in the KMMS validation study sample [13] and explore the role of these factors in perinatal mental health screening and diagnostic outcomes.

## Methods

This project was endorsed by the Kimberley Aboriginal Health Planning Forum Research Subcommittee and has approval from the Western Australian Aboriginal Health Ethics Committee (Project 781) and the Western Australian Country Health Service Human Research Ethics Committee (RGS 2012–23).

### Research design

We used a mixed methods [44] approach to retrospectively analyse a cross-sectional study dataset of completed KMMS assessments [13]. The KMMS assessments were undertaken by female, non-Indigenous midwives and child health nurses during 2013–2014 and form the basis of the KMMS validation for the Kimberley. A detailed account of the original study design and findings have been published elsewhere [13]. The data recorded during Part 2 of the KMMS assessment (the psychosocial yarn [40]) were categorised into psychosocial ‘risk’ and ‘protective’ factors and the characteristics of both categories using a directed qualitative content analysis approach [45]. This included describing protective factors in relation to risk factors where a connection was evident in the data. Second, it involved quantifying risk and protective factors associated with different KMMS risk assessment categories as recorded by the healthcare professional administering the KMMS. The KMMS assessment categories were no risk, low, moderate, or high risk. Third, the relationship between a clinical diagnosis of perinatal depression and/or anxiety and protective and risk factors was explored using statistical analysis.

### Study context and sample

The Kimberley region is a remote and expansive region of Western Australia, beginning approximately 2000 km north of Perth, the state capital. The Kimberley has a total population of about 34,000 people with approximately 42% of all residents identifying as Aboriginal [46]. At a population level, Aboriginal people across the region experience poorer health outcomes and increased disadvantage associated with social determinants of health than non-Aboriginal residents [47].

Our study sample consisted of the files of the 91 Kimberley Aboriginal women who had consented to the KMMS validation study, completed the two-part KMMS and GP mental health assessment [13]. Participants were aged 16–41 years (median 24 years, IQR 20.7–28.9 years) and were receiving their perinatal health care from a Kimberley Aboriginal Community Controlled Health Service (ACCHS) or the Western Australian Country Health Service—Kimberley (WACHS-K). All women were either pregnant (more than 6 weeks gestation) or had a child aged between 7 days and 12 months, and were not known to have been acutely mentally unwell at the time of recruitment. Within the sample, 23 out of 91 women (25%) were clinically diagnosed with depression and/or anxiety [13].

### Data collection and analysis

KMMS Part 2 assessments, i.e. the health professional’s clinical notes of the information provided by the woman during the KMMS ‘yarn’ [40], were transcribed verbatim into individual Microsoft Word documents. These were imported into NVivo 12 (QSR International), and the clinical notes were coded and analysed using a directed qualitative content analysis approach [45]. Qualitative coding and analysis was undertaken by members of the research team (Kimberley Aboriginal Medical Services (KAMS) Aboriginal Research Officer, KAMS KMMS Project Officer, Rural Clinical School of Western Australia (RCSWA) Research Associate and the RCSWA Research Fellow) over eight workshops. The team members were blinded to the participants’ KMMS Part 1 score and KMMS overall risk assessment during the coding and analysis process.

Directed qualitative content coding resulted in three categories of nodes: psychosocial contextual information, psychosocial protective factors and psychosocial risk factors. In subsequent analysis of the data, the three categories were refined and sublevel nodes relating to a particular psychosocial phenomenon were generated; these sublevel nodes included and expanded on the KMMS Part 2 domains (i.e. support, major stressors, self-esteem and anxiety levels, relationships, childhood experiences, social and emotional wellbeing). Information in which no inherent risk or protective factor could be deductively determined was coded as psychosocial context and was excluded from further analysis. We assessed patterns and shared characteristics within the other coded categories to arrive at a rich descriptor of the identified protective factors, risk factors, and protective factors associated with risk factors.

Following data coding, the presence or absence of each protective factor and risk factor for each woman was quantified as a binary variable in Microsoft Excel, along with the presence or absence of a protective factor associated with each risk factor. Using Stata 14 (StataCorp, 2015), these data were amalgamated with additional information from the validation study for statistical analysis including KMMS risk category (no, low, moderate or high risk), and whether there was a clinical diagnosis of perinatal depression and/or anxiety.

For each KMMS risk category, the mean number of total protective and risk factors and percentage of women with most common types of factors were calculated. Trends across categories were assessed using variance-weighted least squares linear regression. The number of protective factors compared to risk factors were summarised for women who did and did not receive a diagnosis of perinatal depression and/or anxiety, with types of factors descriptively investigated for a subset of participants. The associations between a diagnosis and the presence of specific protective and risk factors were analysed using chi-squared tests, and two-tailed Fisher’s exact tests where there were expected cell values < 5. A *p* value of 0.05 was considered statistically significant.

## Findings

### Psychosocial protective factors

For the vast majority of women (88/91), at least one protective factor was identified within their KMMS assessment (mean 4.0, SD 1.7; Table 1). Family-based support was the most frequently recorded psychosocial protective factor among participants overall and was mentioned by 78 women. Support was characterised as practical, emotional, ‘having someone to talk to’ and/or having someone to ‘be with’. Women described general experiences of ‘support’ within the immediate and extended family environment as well as specific family members who provided high levels of support. Mothers were the most commonly mentioned supportive family member, followed by sisters. Family support was generally present for women who lived with or close to family, but was also reported by some who were geographically separated from their family.

**Table 1:** Mean number of protective and risk factors and percentage of women with most common types of factors by KMMS risk category

Protective and risk factors	KMMS risk category				Total (*n* = 91)	*p*-value^a^
	No risk (*n* = 18)	Low (*n* = 45)	Moderate (*n* = 19)	High (*n* = 9)		
Protective factors (PFs)						
Mean total PFs (SD)	4.9 (1.1)	4.3 (1.4)	3.7 (1.5)	1.6 (1.3)	4.0 (1.7)	< 0.001*
Proportion of women with common PFs						
Family (%)	100	87	95	33	86	0.001*
Emotional regulation, self-esteem (%)	94	82	47	11	70	< 0.001*
Healthy lifestyle (%)	78	67	58	22	63	0.008*
Intimate partner relationships (%)	67	73	37	22	59	0.003*
Good childhood (%)	61	51	32	11	45	0.003*
Risk factors (RFs)						
Mean total RFs (SD)	1.1 (1.1)	2.4 (1.3)	3.4 (1.7)	3.8 (1.0)	2.5 (1.6)	< 0.001*
Proportion of women with common RFs						
Adverse childhood experience (%)	28	51	42	67	46	0.1
Grief and loss (%)	17	51	58	22	43	0.3
Anxiety, stress (%)	11	44	58	33	40	0.05
Family (%)	6	29	37	44	27	0.01*
Emotional regulation, self-esteem (%)	6	16	32	67	22	< 0.001*
Intimate partner violence (%)	11	4	37	56	18	< 0.001*
Other intimate partner stressors (%)	6	18	21	22	16	0.2

Based on clear delineation in total percentages (least common included protective factor 45%, most common excluded 18%; least common included risk factor 16%, most common excluded 7%)

KMMS risk category based on assessment by midwife or child health nurse

*KMMS* Kimberley Mum’s Mood Scale, *SD* standard deviation

*Statistically significant linear trend (*p* < 0.05)

^a^Test for linear trend across four KMMS risk categories

While only about a quarter of the women identified their partners as a source of ‘support’, more than half the women in the sample (54/91) described their intimate relationships as ‘good’ and ‘strong’. These women described their partners as ‘caring’, ‘helpful’, taking an active role in fathering and/or excited about the new baby/arrival of the baby. A small number of participants (*n* = 3) identified a history of intimate partner violence which they stated had now ceased; these women identified their partners as ‘good men’.

Most women who identified positive support and connectivity with their family identified themselves as ‘strong’, ‘confident’, managing or coping well, and ‘feeling good’ (64/91). Many of these women reported that they ‘stay away from humbug’ and ‘don’t let things worry me’. Spending time with children and family, going fishing, listening to music, playing sport, gardening, and reading the bible were emphasised by women as ways of enacting self-care and enhancing their sense of wellbeing.

Responsibility for children was another common psychosocial protective factor mentioned by women. Nurturing children, keeping them safe and ensuring children had access to education were common themes. Women expressed a desire to be a role model for their children (both biological and other children in their care). Generally, children were discussed as a normal part of life and a source of both comfort and distraction when life stressors were encountered.

Positive childhood memories and experiences were identified in just under half of KMMS assessments (41/91). In these assessments, childhood is associated with participants feeling ‘safe’, ‘secure’, ‘happy’, being ‘part of an extended and loving family’ and having ‘connection to country’. Women noted a range of different family structures when commenting on their positive childhoods; this included growing up with separated parents, a grandparent or another member of their extended family, and/or growing up with both parents.

Aspects of a healthy lifestyle were mentioned by 57 women. This included not currently or never having used alcohol, cigarettes or marijuana. Women who had given up alcohol and other substances often associated this with a desire to ‘do better’ or ‘set a good example’, one woman noted she was ‘much happier and free now’. Five women talked about broader healthy lifestyle activities such as fishing, walking, taking time out and ‘exercise’. Other sources of psychosocial protective factors less commonly identified were friends, employment and study, religion, and engagement with health professionals.

In univariate analyses of common protective factors, family based support, emotional regulation/self-esteem, having a healthy lifestyle, supportive intimate partner relationships and a good childhood were statistically significantly more likely to be associated with lower KMMS risk (Table 1) and be reported in women who did not have clinical depression and/or anxiety (Table 2).

**Table 2:** Number and percentage of women with different types of protective factors and risk factors, and protective factors associated with risk factors, with or without a diagnosis of depression and/or anxiety in the KMMS validation study

Type of factor	Diagnosed with depression and/or anxiety		Total *n* (%)	*p*-value
	No *n* (%)	Yes *n* (%)		
Protective factor				
Family	64 (94%)	14 (61%)	78 (86%)	< 0.001*
Emotional regulation, self-esteem	56 (83%)	8 (35%)	64 (70%)	< 0.001*
Healthy lifestyle	49 (72%)	8 (35%)	57 (63%)	0.001*
Intimate partner relationships	46 (68%)	8 (35%)	54 (59%)	0.006*
Good childhood	36 (53%)	5 (22%)	41 (45%)	0.009*
Risk factor				
Adverse childhood experience	28 (41%)	14 (61%)	42 (46%)	0.1
Grief and loss	27 (40%)	12 (52%)	39 (43%)	0.3
Anxiety, stress	23 (34%)	13 (57%)	36 (40%)	0.054
Family	15 (22%)	10 (43%)	25 (27%)	0.047*
Emotional regulation, self-esteem	8 (12%)	12 (52%)	20 (22%)	< 0.001*
Intimate partner violence	8 (12%)	8 (35%)	16 (18%)	0.02*
Other intimate partner stressors	12 (18%)	3 (13%)	15 (16%)	0.8
Total women	68 (100%)	23 (100%)	91 (100%)	
Women with each risk factor (RF) who had an associated protective factor (PF)^a^				
PF for adverse childhood experience RF	23 (82%)	8 (57%)	31 (74%)	0.1
PF for grief and loss RF	19 (70%)	3 (25%)	22 (56%)	0.008*
PF for anxiety/stress RF	14 (61%)	10 (77%)	24 (67%)	0.5
PF for family RF	12 (80%)	7 (70%)	19 (76%)	0.7
PF for emotional regulation, self-esteem RF	6 (75%)	6 (50%)	12 (60%)	0.4
PF for intimate partner violence RF	8 (100%)	4 (50%)	12 (75%)	0.08
PF for other intimate partner stressors RF	11 (92%)	3 (100%)	14 (93%)	1.0

Diagnosis by general practitioner following KMMS, blinded to KMMS assessment

*KMMS* Kimberley Mum’s Mood Scale

*Considered statistically significant (*p* < 0.05)

^a^Denominator is the number of women with each risk factor, rather than total women

### Psychosocial risk factors

Only 7/91 women had no reported risk factors; the overall mean number of risk factors was 2.5 (SD 1.6; Table 1).

#### Childhood

Adverse childhood experiences were identified by nearly half of the women (42/91). Family breakdown, having parents that were ‘drinkers’, witnessing domestic violence, childhood abuse (physical and/or sexual), and neglect were the most commonly identified features of adversity. Loss of a parent or a carer was also identified in several of the assessments as a compounding feature to a childhood already shaped by adversity. Approximately, three quarters of participants who mentioned adverse childhood experiences identified an associated protective factor. Primarily, this was being raised by grandparents (mostly a grandmother) or having a grandparent as their ‘safe’ person. Other female members in the family, aunties, or ‘other mothers’ (generally meaning maternal aunts in Western terms) were also identified as having a carer role in the face of family breakdown.

#### Grief and loss

Grief and loss was a pervasive theme with 39 women discussing the impact of loss on their lives. Twenty women (22% of participants) referred to a ‘recent’ death of a significant family member (including, for two women, their child). Within this group, several women identified multiple losses of family members. The death of a loved one often was identified as traumatic (accident, suicide, assault related, long illness with the family member hospitalised far away in Perth). Women often described the anniversary of deaths and the person’s birthday as ‘triggering’.

Most women who identified grief and loss as a risk factor noted talking with family and receiving support from family was a protective factor (22/39). Children were also mentioned as providing a ‘purpose’ and a sense of perspective. Many women reflected on the importance of giving themselves ‘time’ to grieve and being accepting of the grieving process. Around a one quarter of women noted no specific protective factors for grief and loss. The absence of protective factors for this risk was significantly associated with clinical depression and/or anxiety (Table 2).

#### Worry, stress and anxiety

Feelings of ‘worry’, ‘stress,’ or ‘anxiety’ were identified by 40% of women (36/91). Fifteen described these feelings as generalised (‘I think too much’, ‘stressing out at everything’). Other women identified more specific concerns including worry about family members or happenings within their extended family, children (particularly their children’s safety), stress relating to being pregnant (low mood, unplanned pregnancy, lack of support). Other worries/stressors included food security, finances, relationship with partner, insecure living arrangements and involvement with Department for Child Protection.

Two-thirds of women with an anxiety or stress risk factor had an associated protective factor. The majority identified that this involved talking to family and receiving support from family. Most commonly, this support was provided by mothers, aunties and sisters. Several women talked about self-care practices, this included walking, fishing, reading, and being ‘out bush’. Others talked about resiliency: ‘being strong’ and having the ability to ‘cope’ with life. Counselling, church, partners and work were other protective factors identified.

#### Family as stress

While most women identified family as a protective factor, family was also raised by some women as a risk factor for their psychosocial wellbeing (25/91). Eleven women referenced family stress in relation to a family member’s behaviour when intoxicated. Having little or no support from family, arguments and demands from family members, kinship caring responsibilities and family ‘judgement’ were other commonly identified aspects of family-related stress.

Approximately, three quarters of women with a family risk factor identified having protective factors to manage this psychosocial risk. Most commonly, these protective factors emphasised women having a sense of autonomy, ‘good boundaries’ and being able to exercise control over their life. Additionally, women talked about close relationships with other family members as a means of mitigating the stress of a problematic or absent relationship.

#### Intimate partner violence

Sixteen women identified intimate partner violence (IPV). Aspects of IPV included physical abuse, sexual violence, coercion (‘forces me to do things’; ‘forces me to buy drugs’), and jealousy (‘I can’t go anywhere by myself’, ‘doesn’t like [me] spending time with [my] daughter’). One woman identified the violence as ‘worse’ during pregnancy. Problematic drinking and drug taking of partners was mentioned by six women experiencing IPV.

Two women identified the violence occurring in relationships that had recently ended. One of these women stated she is still ‘frightened and shaky’ when talking to her ex-partner but identified the separation as a protective factor against further violence. Ten other women identified family as a protective factor to the IPV. These women characterised support from their family as having someone to talk to or someone to be with, without pressure or expectation. For some women, this included ‘stopping’ with family when her partner is violent or when she can see his behaviour is escalating.

#### Intimate partner stressors

A further 15 women identified a range of other stressors from intimate partners including annoyance, arguing, alcohol and drug use, jealousy, infidelity, relationship breakdown, unstable relationships, ex-partner causing ‘stress’ and communication issues (specific to loss of a child). These women did not report intimate partner violence.

All but one of these women reported associated protective factors, most commonly women adopted self-regulating behaviours (‘Think and talk to myself. I say forget about that—don’t cause a fight’) or physically removed themselves (‘Leave house when partner drinks’) as a means of mitigating the stressor. Other women identified positive communication with their partner and an ability to see the ‘big picture’ and/or an overall sense of satisfaction with the relationship as the means to managing this stressor.

#### Feelings of loneliness, sadness, poor self-esteem and poor emotional regulation.

Twenty women identified feelings of loneliness, sadness, poor self-esteem and emotional dysregulation. Loneliness was identified as a feeling of disconnection as opposed to physical isolation from people and was raised by ten women. Overall women spoke about their desire to ‘stay well and safe for children’ and to be ‘self-motivated and determined’, work and family were other noted protective factors. Two women however spoke about feeling ‘no good’ in themselves, neither woman identified any protective factors.

#### Suicidal ideation, self-harm, history of mental health disorders and substance misuse

Six women discussed recent feelings of suicidal ideation. Five of the six women identified their children and family as the reasons why they would not harm themselves despite the feelings arising in them. One participant had very clear intentions of harming herself and the KMMS notes documented that the primary health care provider arranged for immediate support and intervention.

In addition to the six women above, one woman discussed current self-harming behaviours and a previous suicide attempt. She identified friends as her protective factor. Two women identified a history of depressive disorders. One woman identified a previous history of perinatal depression and anxiety. The other woman identified being on antidepressants and receiving support through counselling services.

Thirty participants identified consuming alcohol, cigarettes and/or other drugs during the perinatal period. Many of these women described attempts to reduce or cease alcohol, cigarettes or marijuana/other drugs either during pregnancy or in the post-natal period.

#### Statistically significant risk factors

Of the more common risk factors, family as stress, lack of emotional regulation/self-esteem, and intimate partner violence were individual significantly associated with higher KMMS risk (Table 1) and having clinical depression and/or anxiety (Table 2).

### Relationship between protective and risk factors, KMMS risk and diagnosis of depression and anxiety

There was a consistent relationship between recorded protective and risk factors and the overall KMMS risk assessment. Women recorded at higher risk less commonly had protective factors recorded (11–33% high risk vs 61–100% no risk) and more commonly had risk factors recorded (22–67% high risk vs 6–28% no risk) than women with lower KMMS-assessed risk (Table 1). Consistent with this, the mean number of protective factors decreased with increasing KMMS risk category (4.9–1.6), with an inverse pattern for risk factors (1.1–3.8) across categories (from no to high risk).

This relationship was also apparent in the independent GP assessments which were blinded to the results of the KMMS. Women diagnosed with clinical depression and/or anxiety had a mean of two protective factors and four risk factors recorded on the KMMS, and frequently had risk factors that did not have a corresponding protective factor. Conversely, those who did not receive a clinical diagnosis of depression and/or anxiety had a mean of approximately four protective factors and two risk factors recorded.

None of the women with protective factors and no risk factors were diagnosed with depression and/or anxiety, while all the women with risk factors and no protective factors were diagnosed (Table 3). Of the women who had an equal or higher number of risk factors compared to protective factors, approximately half were diagnosed with depression and/or anxiety. Of the 50 women who had a greater number of protective factors than risk factors, only four (8%) were diagnosed with mild or moderate anxiety or depression. Notably, all four had a risk factor of grief and loss and three of these did not have an identified protective factor to this risk.

**Table 3:** Summary of relative quantities of protective factors and risk factors for women with or without a diagnosis of depression and/or anxiety in the KMMS validation study

Summary of relative quantity of protective factors compared to risk factors	Depression and/or anxiety		Total
	No	Yes	
Protective factors present; no risk factors	7	0	7
More protective factors than risk factors	46	4	50
Equal or fewer protective factors than risk factors	15	16	31
No protective factors; risk factors present	0	3	3
Total	68	23	91

Diagnosis by general practitioner following KMMS, blinded to KMMS assessment.

*KMMS* Kimberley Mum’s Mood Scale

## Discussion

This study highlights the importance of considering both protective factors and risk factors as well as western biomedical symptoms when screening Aboriginal Australian women’s perinatal mental health. We found that the relative numbers of risk and/or protective factors, the types of factors, and the connections between risk and protective factors were significantly associated with mental health status. Protective factors were observed for the vast majority of the women and appeared to contribute to the absence of anxiety or depression in many women with significant risk factors. The most prominent of these protective factors was positive relationships with family members.

Our findings highlight the particular importance of family as a protective factor for depression and/or anxiety where there is a risk factor of grief and loss (70% vs 25%, *P* = 0.008). These findings about positive family relationships further support the social-ecology framework of resiliency which asserts positive relationships, social cohesion, and community connectedness contribute to resilient individual level functioning [31–33, 35, 48], even in environments characterised by structural risks [49]. The role of positive family connections in supporting Indigenous mental wellness was first demonstrated in a study of 279 Indigenous (but not specifically perinatal) Canadian women [21]. For women with low social support, depression and/or anxiety was significantly higher among those who had experienced childhood adversities versus those who had not. Other studies have also described women’s protective factors having a buffering effect against the detrimental impacts of adverse childhood experiences on mental health [50–52]. In our study, adverse childhood experiences were common (one-third of women) and connections with other family members were described as a protective factor associated with this risk.

Intimate partner violence (IPV) was reported by over one quarter of women in this study. The disclosure of IPV during the screening assessment was significantly associated with a diagnosis of a perinatal mental health disorder, this is consistent with the known adverse impacts of IPV on mental health [53, 54]. We note that Aboriginal women are disproportionately impacted by IPV [55] and often hesitant to discuss IPV with health professionals due to concerns that information may be used to justify the removal of their children by statutory services [56]. Approaches that are culturally safe and strengths based are more likely to be acceptable for Aboriginal women and provide a starting place for support [53, 56] and the KMMS may assist with this.

Our findings advance the argument that traditional mainstream approaches to perinatal mental health screening which focus exclusively on symptoms and risk factors are not sufficient in responding to the needs of Aboriginal Australian women [14, 18, 19]. We have found that while certain risk factors are more indicative of psychological distress the actual significance of a risk factor can only be understood in the unique context of a woman’s overall risk and resiliency. To this effect, one risk factor may significantly and adversely impact an individual woman’s mental health, while for other women, several risk factors may not lead to a clinical mental health diagnosis. This demonstrates the limitations of perinatal mental health screening tools such as the EPDS which predetermine the significance of a risk for women and promote psychosocial investigation and care only when an elevated level of risk has been determined [43, 57, 58].

Aboriginal Australian women have identified that yarning based approaches to screening, which celebrates their resiliency and explores their psychosocial risks is a culturally safe starting point for conversations and screening of their perinatal mental health [13, 42, 43]. The exploration of risk and protective factors in perinatal mental health screening is also closely aligned with the concurrent delivery of psychosocial care by the administering health professionals. In remote and resource poor environments, such as the Kimberley [17], the provision of psychosocial care, including the recognition and validation of a woman’s resiliency, is a desirable, achievable and effective approach to supporting many women at low and moderate risk of mental ill-health [11].

The small sample size of the validation study made it difficult to assess the effect of specific protective factors for risk factors. Further work on both understanding the role of protective factors and accounting for them in perinatal mental health screening for Aboriginal Australian women and their global Indigenous counterparts is recommended. The directed qualitative content analysis was based on a relatively large number of participants which was independently collected by local clinical staff who generally knew the participant. However, these data were somewhat limited by the extent to which information was recorded, and therefore, lacks the thickness of a phenomenological or first person approach to gathering data.

### Conclusion

This paper highlights the importance of protective factors in supporting the mental health of Indigenous peoples across a range of colonised settings, who experience shared characteristics of structural risk. Our study demonstrated that the presence of protective factors was associated with the absence of a clinical diagnosis of depression and/or anxiety. These protective factors can be understood as expressions of resiliency. We recommend that screening tools which include exploration of protective factors are more broadly adopted in mental health, including perinatal mental health, screening for Indigenous peoples.

## Data Availability

Data cannot be shared publicly because of the ethical restrictions and data sovereignty considerations involved in working with a small population of Aboriginal people in Western Australia. Indigenous data sovereignty is defined as the right of Indigenous peoples to determine the means of collection, access, analysis, dissemination and reuse of data pertaining to the Indigenous peoples from whom it relates or was derived from. As such, the minimal dataset will be made available upon request for researchers who meet the criteria for access to confidential data. Interested researchers should contact Emma Carlin at Emma.Carlin@rcswa.edu.au for data requests.
